# Sequence Control in Sulphur‐Containing Ring‐Opening Co‐ and Terpolymerisations

**DOI:** 10.1002/anie.202507243

**Published:** 2025-05-30

**Authors:** Bhargav R. Manjunatha, Cesare Gallizioli, Christoph Fornacon‐Wood, Jenny Stephan, Merlin R. Stühler, Alex J. Plajer

**Affiliations:** ^1^ Makromolekulare Chemie Universität Bayreuth Universitätsstraße 30 95447 Bayreuth Germany; ^2^ Intitut für Chemie und Biochemie Freie Universität Berlin Fabeckstraße 34/36 14195 Berlin Germany; ^3^ Bayrisches Polymer Institut (BPI) Universität Bayreuth Universitätsstraße 30 95447 Bayreuth Germany

**Keywords:** Polymerisation Catalysis, Ring‐Opening Polymerisation, Sulphur‐Containing Polymers

## Abstract

Sulphur‐containing polymers uniquely expand the catalogue of accessible material properties compared to current commodity materials, yet their synthesis remains underdeveloped. Combining sulphur‐ and oxygen‐containing monomers in ring‐opening copolymerisation leads to a reshuffling reaction of the sulphur centres, which has been challenging to control — a central task for reliably tailoring properties. However, very recent methodologies have emerged that not only suppress but also utilise this phenomenon to precisely access sulphur‐containing polymer structures. These structures can exhibit improved degradability, chemical recyclability, refractive indices and crystallinity compared to their all‐oxygen analogues. Furthermore, underutilised and even entirely untapped monomer feedstocks can become accessible as a result. This minireview aims to provide a roadmap of the tools currently available to selectively access sulphur‐containing co‐ and terpolymer structures to enable emerging applications that leverage the chemistry of sulphur.

## Graphical abstract

Sulphur‐containing polymers offer unique avenues for degradability, recyclability and optical performance—but their synthesis is often plagued by side reactions. This minireview outlines recent strategies to control or harness O/S scrambling in ring‐opening co‐ and terpolymerisations, enabling precise sequence control. Emerging applications in dynamic materials, self‐assembly, and sustainable plastics are highlighted.

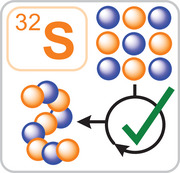

## 1 Introduction

**Figure 1:**
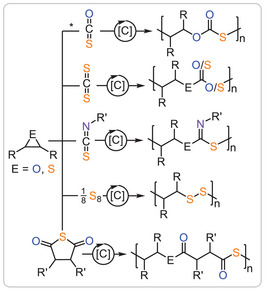
Examples of sulphur‐containing ROCOP monomer combinations based on epoxides (E═O) and thiiranes (E═S). *only reported for epoxides. [C] = catalyst.

Associated with the underlying chemistry of sulphur, sulphur‐containing polymers exhibit unique material properties.^[^ ^1^ ^]^ For example, the chemically soft lone pair of sulphur centres allows these polymers to coordinate transition metals, which can be utilised to purify contaminated water.^[^ ^2^, ^3^ ^]^ Furthermore, the greater lability of sulphur's covalent bonds, compared to elements from the second period (typically used in commodity polymers), not only makes these bonds easier to break but also to exchange. This renders sulphur‐based materials capable of forming covalent adaptable networks, which can be thermally reshaped without added catalysts.^[^ ^4^ ^]^ The ability to oxidise sulphur not only offers potential for chain cleavage but also allows alteration of polymer polarity and solubility.^[^ ^5^ ^]^ Additionally, sulphur's increased molar refractivity imparts higher refractive indices to these polymers, useful, for example, in reducing the thickness of polymer lenses.^[^ ^6^, ^7^ ^]^ Sulphur‐containing polymers can also exhibit increased semi‐crystallinity compared to their all‐oxygen analogues, resulting in improved mechanical performance,^[^ ^8^ ^]^ while sulphur's greater angular flexibility can reduce ring strain in sulphur‐containing heterocycles. As a direct consequence of the latter, polymers derived from the ring‐opening polymerisation of such heterocycles exhibit decreased ceiling temperatures compared to their all‐oxygen analogues, thus facilitating depolymerisation — an important feature in chemical recycling of polymers back to their monomers as well as dynamic and responsive behaviour.^[^ ^9^, ^10^, ^11^, ^12^, ^13^, ^14^, ^15^, ^16^, ^17^, ^18^ ^]^ Taken together, it is clear that incorporating sulphur centers into polymer chains not only enables unique applications but also addresses sustainability challenges that current commodity materials face. Therefore, much effort has focused on developing synthetic access to these materials beyond traditional polycondensation and vulcanization methodologies. In this regard, impressive progress has been made in the fields of inverse vulcanization and ring‐opening polymerisation.^[^ ^19^, ^20^, ^21^ ^]^ Much progress has also been made in the field of ring‐opening copolymerisation (ROCOP), where a strained heterocycle is copolymerised with a second monomer. Figure 1 summarises known monomer combinations and the derived polymer structures based on epoxides and thiiranes that can be selectively assembled by a catalyst C known in ROCOP to date.

The notable advantages of ROCOP include, on one hand, the wide variety of polymer structures that are accessible, and on the other, the diverse monomers that can be used. These monomers include not only industrially relevant epoxides (such as propylene and ethylene oxide) but also many functional derivatives developed in the context of epoxide ring‐opening polymerisation.^[^ ^22^ ^]^ Moreover, ROCOP enables the formation of copolymers from monomers that do not form stable homopolymers, which is particularly relevant in the context of polymerising elemental sulphur, a waste product of the petrochemical industry. While many sulphur‐containing ROCOPs are known (previously summarised by one of the authors in 2021^[^ ^23^ ^]^), they can be plagued by numerous side reactions that negatively impact polymer yield, regularity of the polymer microstructure and molecular weight. These side reactions occur to a much greater extent for some monomers than for others. Recent years have seen significant progress in understanding when and why these side reactions occur. These developments not only allowed to avoid these and thereby optimise material performances, but most importantly even utilizing them to develop new classes of sequence selective terpolymerisations. Hence, this minireview aims not only to highlight recent progress in sulphur‐containing ROCOP but also to shed light on the factors that govern selectivity, enabling the design of new polymerisation methodologies for degradable and functional sulphur‐based materials.

## 2 The Oxygen–Sulphur Exchange Reaction in Epoxide ROCOP

**Figure 2:**
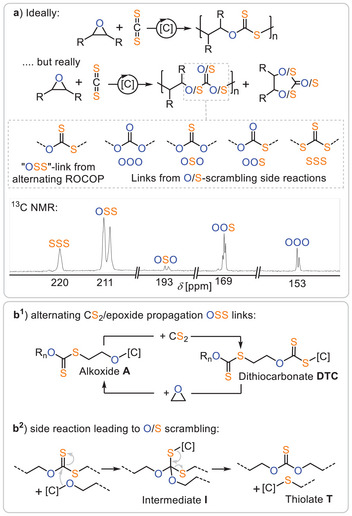
a) Product distribution observed in the reaction of CS_2_ with epoxides and various catalysts [C]; the inlay shows zoom into the quaternary carbonyl R_2_C═O/S region of a typical ^13^C NMR spectrum of an isolated CS_2_/epoxide copolymer. Reaction pathways leading to (b^1^) alternating OSS links as well as (b^2^) O/S scrambled links. Figure adapted from ref. [24] under CC BY 4.0.

Considering Figure 1, one might expect the ROCOP of CS₂ and epoxides to yield polydithiocarbonates featuring ‐O‐(C = S)‐S‐ (OSS) linkages (Figure 2a), with the oxygen atoms coming from the epoxide and the sulphur atoms coming from carbon disulfide. However, CS₂/epoxide ROCOP has proven to be more complex than anticipated. Since the first report on carbon disulfide/epoxide copolymerisation, linkages other than the expected OSS from alternating copolymerisation have been observed. Adachi and coworkers first employed Et₂Zn‐based catalysts in CS₂/propylene oxide (PO) ROCOP and observed, via IR spectroscopy, significant amounts of R₂C═O groups, such as ─O─(C═O)─O─ (OOO) and ─O─(C═O)─S─ (OOS) linkages, as part of the polymer.^[^ ^24^ ^]^ From this point onward, the formation of linkages other than OSS will be referred to as O/S scrambling. Thereafter, both heterogenous and bicomponent transition metal and borane‐based catalysts have been employed for the copolymerisation of CS_2_ for a range of different epoxides and links from O/S scrambling were always observed in dominant proportions. As part of these explorations Darensbourg, Zhang and others formulated different mechanistic hypotheses on the origin of the scrambling process which all involve nucleophilic attack of an OSS link.^[^ ^25^, ^26^, ^27^, ^28^ ^]^ Although, historically many different nucleophiles (epoxides monomers, cocatalysts, chain ends) have been proposed to lead to this pathway, the following most robust mechanistic hypothesis emerged in recent years.

**Figure 3:**
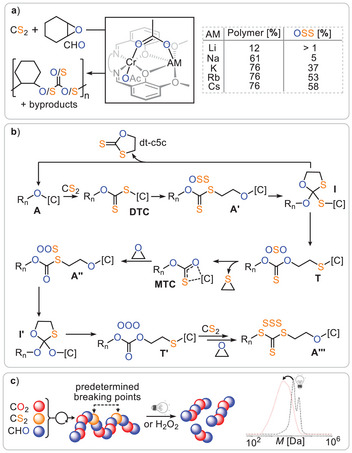
a) CS_2_/CHO ROCOP catalysed by a series of heterobimetallic CrAM catalysts.^[^ ^29^ ^]^ b) DFT modelled propagation pathway explaining O/S scrambling for ethylene oxide EO to reduce computational cost. c) Statistical CO_2_/CS_2_/CHO terpolymerisation installing predetermined breaking points into polycarbonate; overlaid GPC traces before and after irradiation. Figure adapted from ref. [24] under CC BY 4.0.

As shown in Figure 2b, alternating CS₂ and epoxide insertion produces catalyst‐bound dithiocarbonates **DTC** and alkoxides **A** through alternating insertion, creating the OSS links. However, if an alkoxide **A** reacts with an OSS link from, for example, another polymer chain, a tetrahedral intermediate **I** forms, which then collapses to generate a thiolate **T** and an OSO link. In this reaction sequence, the polymer link is effectively enriched in oxygen, transforming OSS to OSO. Propagation of the thiolate by CS₂ insertion results in a sulphur‐enriched ─S─(C═S)─S─ (SSS) link. Although, this hypothesis provides a solid starting point, it does not explain all observed linkages. To address this, our group recently reported a comprehensive study on CS₂/cyclohexene oxide (CHO) ROCOP using a series of heterobimetallic CrAM catalysts (AM = Li, Na, K, Rb, Cs), which completed the mechanistic understanding of O/S scrambling (Figure 3a).^[^ ^29^ ^]^

Moving down the periodic table from Li to Cs revealed that an improvement in polymer selectivity (versus cyclic dithiocarbonate dt‐c5c) is associated with increased OSS selectivity (i.e., less O/S scrambling), supporting the idea that the reaction pathways leading to dt‐c5c and O/S scrambled links are mechanistically related. Importantly, in the all‐oxygen ROCOP, cyclic byproducts are known to form via so‐called backbiting, in which alkoxide intermediates react with polymer links directly adjacent to the chain end.^[^ ^30^ ^]^ Model reactions revealed that the reaction between metal alkoxides and organic (di)thiocarbonates results in the formation of oxygen‐enriched metal carbonates and thiirane byproducts, which are sulphur‐containing analogues of epoxides. The latter could also be identified as byproducts of the CS₂/CHO ROCOP, providing key information to complete the mechanistic picture for the O/S scrambling mechanism shown in Figure 3b.

The key extension of the previous mechanistic hypothesis is that alkoxides **A’** formed following epoxide insertion from **DTC** react with dithiocarbonate OSS links directly adjacent to the chain end to form a cyclic version of intermediate **I**. This then collapses into a thiolate **T** that sits adjacent to an OSO. Furthermore, catalyst‐bound thiolates can eliminate thiiranes to form catalyst‐bound thiocarbonates **MTC** that propagate and undergo analogous O/S scrambling at the chain end (forming thiolate **T’** sitting adjacent to an OOO link). O/S scrambling is thermodynamically favourable, as indicated by density functional theory, likely due to the accumulation of negative charge on the larger sulphur centers. In the context of O/S scrambling, it should be noted that, Zhang reported for COS/PO ROCOP that scrambling can also stem from catalysed COS hydrolysis into CO₂, which itself serves as a monomer to produce, for example, all‐oxygen carbonate (OOO) linkages, highlighting the potential for these pathways to be also at play in CS_2_ ROCOP.^[^ ^31^ ^]^ Due to the mechanistic similarity of all heteroallene/epoxide copolymerisations, CS₂/CHO ROCOP could be combined with CO₂/CHO ROCOP in a CO₂/CS₂/CHO terpolymerisation (Figure 3c). In this case, OOO linkages predominantly form due to kinetically favoured CO₂ insertion. Nevertheless, the scrambled sulphur‐containing linkages rendered the polymer oxidatively and photochemically labile—degradability pathways that are not accessible to the all‐oxygen polycarbonate analogue. This highlights a larger concept: introducing a few sulphur centers into oxygen‐containing polymers can act as predetermined breaking points to enhance degradability.

**Figure 4:**
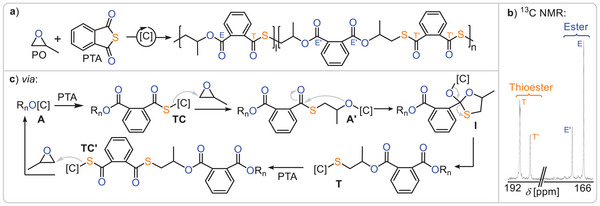
a) Product distribution of PTA/epoxide. b) Zoom into the quaternary carbonyl R_2_C═O region of a typical ^13^C NMR spectrum of an isolated PTA/epoxide copolymer. c) Mechanistic hypothesis explaining the resonance distribution observed in the ^13^C NMR spectrum. Figure adapted from ref. [32] under CC BY 3.0.

An alternative approach to sulphur‐containing copolymers from epoxides is the ring opening copolymerisation with cyclic phthalic thioanhydride (PTA) producing semiaromatic materials comprising ester and thioester links (Figure 4). Although alternating propagation would precisely yield ester‐*alt*‐thioester, Ren and coworkers also observed the formation of diester and dithioester links in the polymer chain of PTA/PO copolymer from bicomponent catalysis (*M*_n,max_ *_._* = 60 kg mol^−1^, *Đ* = 1.4) based on the commercial SalCyCr(III)Cl/ nitridobis(triphenylphosphan)chlorid (PPNCl) catalyst pair.^[^ ^33^ ^]^ This observation is reminiscent of O/S‐scrambling in CS_2_ ROCOP likewise producing linkages enriched (dithioester) and depleted (diester) in sulphur compared to the perfect sequence. Figure 4c shows the mechanistic hypothesis developed later by our group (see below) featuring an O/S‐scrambling mechanism at the propagating chain end via a cyclic intermediate **I** that transforms an alkoxide chain end **A’** into a thiolate chain end **T**. At higher reaction temperatures, other erroneous links that presumably form through thiirane elimination pathways in analogy to CS_2_/epoxide ROCOP were also observed. Yet low reaction temperature and catalysts based on ligand scaffolds with tethered cocatalysts could suppress these side reactions for PTA/PO ROCOP (*M*_n,max_ *_._* = 50 kg mol^−1^, *Đ* = 1.2) and also yielded strictly alternating ester‐*alt*‐thioester from a range of other epoxides.^[^ ^34^ ^]^

**Figure 5:**
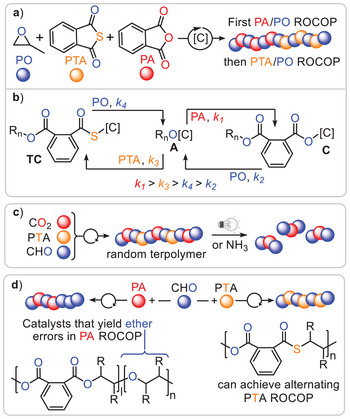
a) Blockpolymerisation from mixtures of PA, PTA and PO. b) Relative rate of individual insertion events explaining the blockpolymer formation. c) Random terpolymer formation CO_2_, PTA and CHO. d) Comparing PA with PTA ROCOP employing the same family of heterobimetallic Al(III)M catalysts (M = (Earth)alkalimetal).

**Figure 6:**
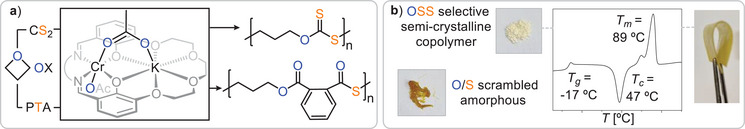
a) Selective OX ROCOPs based on Oxetane (OX). b) Sequence selective CS_2_/OX ROCOP resulting in semi‐crystalline materials whereas O/S scrambling yields an amorphous material. Figure adapted from ref. [29] under CC BY 4.0.

Li and coworkers reported a bicomponent catalyst based on an Al(III) complex featuring a bipyridine‐based tetradentate N,N,O,O ligand system that also suppressed O/S scrambling in PTA/PO polymerisation (*M*_n,max_ *_._* = 54 kg mol^−1^, *Đ* = 1.4), allowing the use of a wide range of epoxides.^[^ ^35^, ^36^ ^]^ Mixtures of PTA, phthalic anhydride (PA), and PO resulted in block copolymer formation, where the PA/PO polyester block formed before the PTA/PO block (Figure 5a), even though the latter reaction occurred faster in the stand‐alone ROCOP. This observation can be rationalised by the fact that alkoxide intermediates (which both polymerisations share from PO ring‐opening) chemoselectively insert into PA over PTA due to the higher partial positive charge on the carbonyl center of PA, making it more susceptible to nucleophilic attack by alkoxide intermediates (Figure 6a).

In our own research, we terpolymerised PTA and CO₂ with CHO using heterobimetallic catalysts and observed statistical terpolymer formation in which the links from PTA/CHO deviated from strict ester‐*alt*‐thioester alternation, resulting in more ester than thioester links.^[^ ^37^ ^]^ We identified this deviation to occur via a thiirane elimination pathway, which led to oxygen‐enriched terpolymers. Nevertheless, the randomly distributed thioester links in the polymer main chain enhanced degradability upon UV irradiation as well as through aminolysis with ammonia. Moving from Chromium(III)‐based heterobimetallics to Aluminum(III)‐based catalysts within the same ligand scaffold led to at least a 10‐fold activity enhancement in PTA/CHO ROCOP.^[^ ^38^ ^]^ Employing 4‐vinyl‐substituted CHO allowed us to achieve a maximum *M*_n_ of 67.2 kDa (*Đ* = 1.4). Interestingly, comparative assessment of alkali, alkaline earth metals and lanthanum as the second metal in PA and PTA/CHO ROCOP revealed that some catalysts produced ether defects in the polyester from PA ROCOP, resulting from erroneous CHO homopropagation. In contrast, the very same catalysts were able to achieve alternating PTA/CHO ROCOP with minimal amounts of scrambling. While the underlying reasons remain to be fully understood, it appears that moving to sulphur‐containing monomers provides intrinsic sequence‐selectivity benefits.

## 3 Reducing O/S Scrambling in Oxetane ROCOP

Although SalcyCrCl‐based catalysts yielded heavily O/S scrambled copolymers alongside cyclic six‐membered trithiocarbonate byproducts in the first attempt at CS₂/oxetane (OX) ROCOP, a heterobimetallic analogue combining a SalCy pocket for Cr(III) coordination with a 18‐crown‐6 pocket for K coordination produced a copolymer with up to 96% OSS linkages and 99% polymer selectivity (see Figure 6).^[^ ^27^, ^39^ ^]^ This led to an up to 7‐fold improvement in the maximum obtainable molecular weight (*M*_n_ = 100 kg mol^−1^, *Đ* = 1.6) over the previous state of the art catalysts. Both, the choice of metals and fixing both metals within the same ligand scaffold were critical in achieving the highest selectivity. The report further emphasised the importance of selective catalysis to enhance material properties, as the polymer microstructure strongly influenced the thermal and mechanical properties of the materials. While the O/S scrambled polymers were amorphous with a *T*_g_ of −29 °C, the polymers with high OSS selectivity were semicrystalline with a *T*_m_ of 90 °C (and a *T*_g_ of −17 °C). Thus, sequence‐controlled polymers can be processed into flexible films, whereas scrambled polymers cannot. The semicrystallinity is particularly noteworthy, as the all‐oxygen counterpart polytrimethylene carbonate (with all OOO linkages) is also an amorphous polymer, highlighting that replacing oxygen with sulphur centers in otherwise isostructural polymers can lead to improved thermal and mechanical properties.^[^ ^40^, ^41^ ^]^ Furthermore, we found that, compared to all‐oxygen polycarbonates, polytrimethylene dithiocarbonate undergoes more facile depolymerisation due to the decreased ring strain exhibited by sulphur‐containing heterocycles. In parallel, Zhang reported that triethylborane/PPNCl organocatalysts mediate quantitative polymer formation and up to 98% sequence selective CS_2_/OX ROCOP at room temperature (*M*_n,max._ = 40 kg mol^−1^, *Đ* = 1.7).^[^ ^42^ ^]^ Computational studies indicated that while CS_2_ insertion only requires the aid of one borane, both the catalyst bound dithiocarbonate chain end and the incoming oxetane monomer require borane activation in the rate determining step of the polymerisation which could be confirmed experimentally as such that a 2:1 BEt_3_/PPNCl ratio showed superior activity to a 1:1 ratio.

Moving to the synthesis of polythioesters, our group reported that heterobimetallic CrAM catalysts (AM═Na, K, Rb), based on the macrocyclic ligand scaffold mentioned above, deliver alternating poly(ester‐*alt*‐thioesters) from PTA and OX.^[^ ^43^ ^]^ In contrast to their performance in CS₂/OX ROCOP, all alkali metals, as well as catalysts where both metals are coordinated by different ligand scaffolds, produced perfectly alternating semicrystalline (*T*_m_ = 88 °C) copolymers. Higher maximum molecular weights (*M*_n_ *_,_*_max_ *_._* = 139 kg mol^−1^, *Đ* = 1.3) could be achieved than previously reported for PTA ROCOPs alltogether.

Subsequently, we confirmed that PTA/OX ROCOP is highly catalyst‐tolerant, as all investigated systems—including bicomponent SalCyMCl (M═Cr(III), Al(III)) or BEt₃ catalysts in combination with PPNCl cocatalysts—yield perfectly alternating copolymers.^[^ ^32^ ^]^ By systematically comparing PTA ROCOP with oxetanes, epoxides and thiiranes using a heterobimetallic CrK catalyst, we identified factors that lead to high sequence selectivity in OX ROCOP. Backbiting reactions occur to a lesser extent when heterocycles are used that produce primary alkoxide chain ends upon ring opening. This means that ethylene oxide, ethylene sulfide, oxetane and 3,3′‐disubstituted oxetanes are particularly suited monomers in sulphur‐containing ROCOP. Computational studies revealed that for epoxides, PTA insertion is exergonic for ring‐opening by primary alkoxides, while it is endergonic for secondary alkoxides. Furthermore, bicyclic epoxides kinetically disfavour backbiting by leading to strained intermediates. Nevertheless, the ROCOP must be terminated in a timely manner, ideally before complete monomer conversion, as intermolecular scrambling pathways can disrupt polymer chains even after the polymerisation has finished. Capitalizing on this circumstance, we then used BEt₃ in conjunction with KOAc to achieve selective PTA/OX ROCOP, with performance further boosted by coordinating K with 18‐crown‐6.^[^ ^44^ ^]^

## 4 Reducing O/S Scrambling in Isothio‐Cyanate ROCOP

Some heteroallenes are intrinsically resistant to O/S scrambling, such as carbonyl sulfide (COS), for which many highly selective copolymerisations have been developed and reviewed in the past.^[^ ^45^ ^]^ However, COS is a toxic gas and, for example, is not commercially available in Europe. In recent years, isothiocyanates, valuable building blocks in thiirane ROCOP as for example demonstrated by *Wu*,^[^ ^46^, ^47^ ^]^ have emerged as suitable monomers that, like COS, undergo less O/S scrambling with epoxides and oxetanes.

**Figure 7:**
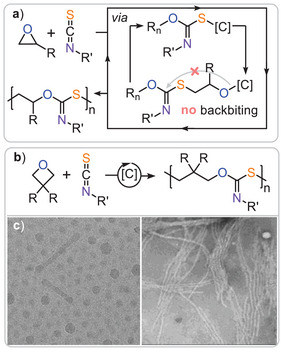
a) Isothiocyanate/epoxide ROCOP selectively yielding thioimidocarbonates without the occurrence of O/S scrambling. b) Isothiocyanate/oxetane ROCOP and c) self‐assembled nanostructured by on derived diblock copolymers. Figures adapted from ref. [50] under CC BY 4.0.

Both simple lithium salts and BEt₃‐based bicomponent catalysts yield poly(monothioimidocarbonates) featuring ─O─C(═NR)─S─ thioimidocarbonate links instead of the theoretically possible ─O─C(═S)─NR─ thionourethane links (Figure 7).^[^ ^48^, ^49^ ^]^ This occurs on a kinetic basis, as the formation of thionourethane links is thermodynamically more stable but associated with higher insertion barriers during the epoxide ring‐opening step. Maximum molecular weights of *M*_n,max_ *_._* = 59 kg mol^−1^ (*Đ* = 1.1) were achieved from PhNCS and PO, although a variety of other epoxides, as well as different aromatic and aliphatic RNCS, were also successfully copolymerised, yielding amorphous copolymers with glass transition temperatures ranging from −36 °C to 120 °C. Some of the resulting poly(thioimidocarbonates) (EtNCS‐co‐CHO) could be used as positive resists for electron beam lithography, outperforming commercially used poly(methyl methacrylate) in terms of sensitivity (130 versus 180 µC cm^−^^2^). These materials could also be utilised in the construction of two‐dimensional photonic crystals. Furthermore, PhNCS/PO ROCOP could be combined with PA/PO ROCOP, where ternary mixtures of PA, PhNCS and PO resulted in block copolymer formation, similar to the previously discussed PA/PTA/PO terpolymerisation. In this process, PA/PO ROCOP occurs first, forming a polyester block, followed by PhNCS/PO ROCOP, which forms imidothiocarbonate blocks.

In our own research, we reported on the ROCOP of RNCS with oxetanes under heterobimetallic CrRb catalysis, which proceeded without O/S scrambling, though minor amounts of the kinetic thionourethane ─O─C(═S)─NPh─ links were formed alongside the dominant (>90%) thioimidocarbonates ─O─C(═NPh)─S─.^[^ ^50^ ^]^ High maximum molecular weights of *M*_n,max_ *_._* = 124 kg mol^−1^ (*Đ* = 1.7) were achieved for PhNCS/OX, and this monomer combination also exhibited high melting points of 178 °C. While aryl‐substituted RNCS yielded semi‐crystalline materials, the aliphatic cyclohexyl analogue, produced amorphous materials when copolymerised with oxetanes. PhNCS generally resulted in narrower molecular weight distributions than CS₂ under similar conditions with oxetanes, making this monomer combination particularly useful for constructing block copolymers. By adding mPEG‐OH macroinitiators as chain transfer agents, amphiphilic block copolymers were formed, which could be assembled into various morphologies (micelles, worms, cylinders, platelets) through (crystallisation‐driven) self‐assembly. Analogous block polymers could be formed from aryl substituted isothiocyanates and also PTA as a comonomer forming self‐assembled structures in water in which transition metal salts and complex fragments could be coordinated by the sulphur‐containing cores.^[^ ^51^ ^]^ In contrast, CS_2_/oxetane ROCOP in the presence of chain transfer agents led to substantial scrambling which, in light of results in oxygenated ROCOP implies that scrambling also occurs via off catalyst pathways via initial decoordination of the chain end.^[^ ^52^ ^]^

## 5 Controlling O/S Exchange in Sequence Selective Terpolymerisation

**Figure 8:**
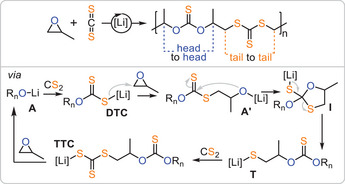
Lithium catalysed CS_2_/PO copolymerisation delivering a poly(OSO‐*alt*‐SSS) sequence.

Although O/S scrambling has mostly been considered a complication, it also presents an opportunity by introducing chemical complexity that, if controlled, could provide access to unique polymer sequences. In this context, a landmark report by *Werner* and *Komber* demonstrated that lithium alkoxide catalysts deliver polymers consisting of alternating OSO and SSS links from CS₂ and PO (Figure 8).^[^ ^28^ ^]^ In these polymers, the OSO links are connected to the CHR₃ “head” positions of the ring‐opened PO, while the SSS links are connected to the CH₂R₂ “tail” positions of the ring‐opened PO. These links are formed with up to 92% sequence selectivity, reaching molecular weights up to *M*_n_ = 109 kDa (*Đ* = 1.8). The formation of these links can be rationalised by a propagation pathway in which “tail‐selective” PO ring‐opening by **DTC** occurs, forming a secondary alkoxide **A’** that rapidly backbites into an adjacent OSS link to create the cyclic intermediate **I**. This intermediate then collapses into a primary thiolate **T**, which propagates by inserting CS₂ and forming a metal‐bound trithiocarbonate **TTC**, which tail‐selectively inserts PO to complete the cycle. Essentially, this report demonstrated that simple lithium salt catalysts can mediate O/S exchange in a highly selective manner—a feat that other catalysts could not achieve—making it all the more remarkable given their simplicity.

**Figure 9:**
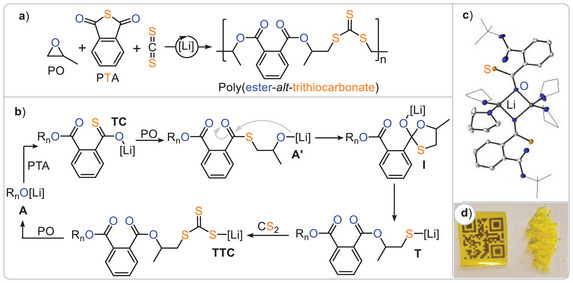
a) Lithium catalysed PTA/CS_2_/PO ROTERP delivering poly(ester‐*alt*‐trithiocarbonates) in a head‐to‐head‐*alt*‐tail‐to‐tail regioselectivity. b) Experimentally and computationally substantiated mechanistic hypothesis. c) Crystal structure of the suspected lithium thiocarboxylate resting state. d) Photograph of the obtained polymer as a free‐standing film and powder. Figure adapted from ref. [53] under CC BY 3.0.

Building upon the concept of accessing unique polymer sequences through O/S exchange, our group serendipitously discovered that LiX (X═OCH_2_Ph, N(SiMe₃)₂) achieves the formation of poly(ester‐*alt*‐trithiocarbonates) from mixtures of PTA, CS₂ and PO (Figure 9) with up to 98% sequence selectivity and high maximum molecular weights (*M*_n,max_ *_._* = 111 kg mol^−1^, *Đ* = 1.8). These polymers are amorphous (*T*_g_ = 34 °C), yellow (due to the C═S chromophore) and exhibit good refractive indices around 1.6.^[^ ^53^ ^]^ Resembling *Werner*’s and *Komber*’s copolymer, the oxygen‐rich ester groups are connected to the CHR₃ head positions of the ring‐opened PO, while the sulphur‐rich trithiocarbonate groups are connected to the CH₂R₂ tail positions, forming a head‐to‐head‐*alt*‐tail‐to‐tail microstructure. The mechanism, as shown in Figure 8, necessitates near‐complete O/S scrambling of lithium alkoxides adjacent to thioesters, as well as selective CS₂ over PTA insertion by lithium thiolates **T** and selective PTA over CS₂ insertion of lithium alkoxides **A** adjacent to trithiocarbonates. A combined experimental and computational study demonstrated that although both PTA and CS₂ can be inserted by both lithium alkoxide **A** and thiolate **T**, selective insertion occurs on a kinetic basis.^[^ ^54^ ^]^ Alkoxide **A** inserts PTA orders of magnitude faster than CS₂, while the reverse is true for thiolate **T**. Furthermore, lithium acts as a true catalyst rather than a spectator counterion, as attempts to employ sodium or potassium instead led to poor performance. This can be rationalised by single crystal XRD analysis of model thiocarboxylate intermediates **TC**, identified as the catalytic resting state, which revealed dimeric structures where the sulphur centers remain uncoordinated and accessible for propagation. Additionally, the high oxophilicity and flexible coordination chemistry of lithium enable monomeric, dimeric and charge‐separated transition states, all active in various propagation steps, explaining the unique role of the lithium catalyst. As it turned out a series of monomer combinations and blockpolymerisations on the basis of this sequence selective ring‐opening terpolymerisation (ROTERP) could be identified.

**Figure 10:**
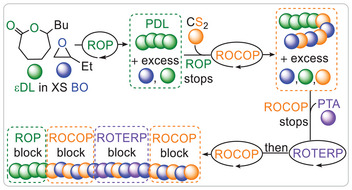
One‐pot block polymerisation combining ROP, ROCOP, and ROTERP for multiblock polymer synthesis. Figure adapted from ref. [56] under CC BY 4.0.

Similarly, mixtures of PTA, PhNCS, and butylene oxide (BO) selectively formed poly(ester‐*alt*‐dithioimidocarbonates) (*M*_n,max_ *_._* = 14 kg mol^−1^, Đ = 1.5), featuring and ─S─(C═NPh)─S─ link albeit with a reduced sequence selectivity of ca. 90%. These new sequence‐selective ROTERPs could then be employed for the design of one‐pot block polymerisation.^[^ ^55^, ^56^ ^]^ For example, lithium alkoxide‐catalysed ε‐decalactone (εDL) polymerisation in BO as a solvent could be continued with CS₂/BO ROCOP by the addition of CS₂, followed by PTA/CS₂/BO ROTERP with the subsequent addition of PTA, which switched back to ROCOP once PTA was depleted, ultimately forming tetrablock polymers (Figure 10).

**Figure 11:**
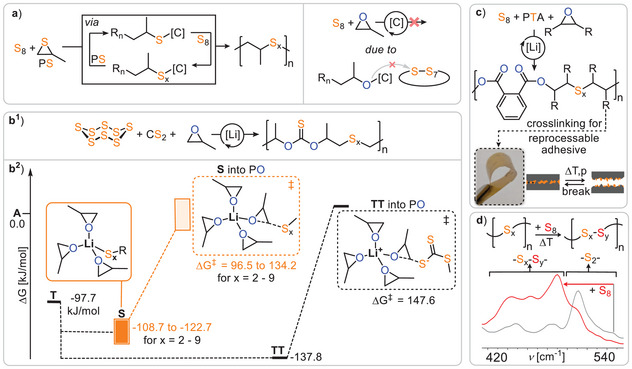
a) S_8_/thiirane ROCOP involving catalyst bound thiolate and polysulfide intermediates. Hypothetical S_8_/epoxide ROCOP prevented by thermodynamically unfavourable alkoxide insertion into ‐S─S‐ bond. b^1^) S_8_/CS_2_/PO ROTERP as well as (b^2^) energetics of selectivity determining step. c) S_8_/PTA/PO ROTERP and example applications of crosslinked derivatives as thermally reprocessable adhesives. d) Thermally activated S_8_ insertion into polyester backbone with associated Raman spectra of the ‐S_x_‐ region before and after S_8_ insertion. Figures adapted from ref. [59] under CC BY 4.0. under and ref. [60]

Importantly in the above mentioned ROTERPs, the monomers which are combined in the terpolymerisation are mutually compatible meaning that the ROTERP effectively represents the combination of two ROCOPs, e.g. CS_2_/PTA/PO ROCOP combining CS_2_/PO and PTA/PO ROCOP which can be both achieved individually by the lithium catalyst. Going beyond that, we hypothesised that the lithium thiolate intermediates generated during O/S exchange could facilitate the incorporation of monomers in terpolymerisation with epoxides that otherwise wouldn't undergo ROCOP with epoxides. In this regard, elemental sulphur S_8_, a megaton waste product from the oil refining process, serves as a suitable ROCOP monomer when combined with thiiranes but not with epoxides (Figure 11a).^[^ ^57^, ^58^ ^]^ As originally demonstrated by *Penczek* and *Duda*, and refined by *Lu* and *Ren*, thiolate intermediates generated from the ring‐opening of thiiranes can activate elemental sulphur, ultimately forming polysulfide polymers of the form [CR₂CR₂S_x_]_n_, where the sulphur rank x can be controlled by adjusting the amount of initially supplied sulphur. Since thiolates are also generated during O/S scrambling, they should, in principle, allow for the activation of S_8_ as well. Realizing this concept, we reported the lithium‐catalysed ROTERP of S_8_ with CS_2_ and epoxides, producing poly(OSO‐*alt*‐S_x_) (*M*_n,max_ *_._* = 30 kg mol^−1^, *Đ* = 1.5) with up to 99% sequence and 90% polymer selectivity (Figure 11b^1^).^[^ ^59^ ^]^ While S_8_ ROCOP requires expensive thiirane comonomers, ROTERP enables polymer formation of S_8_ using industrially available epoxides and CS_2_. Carbon disulfide is 84 wt% sulphur and obtained from elemental sulphur waste itself. Compared to the parent reaction of CS_2_ with epoxides, the addition of sulphur led to increased polymer yields, particularly at higher reaction temperatures, where, without S_8_, no polymer and only cyclic dithiocarbonate was obtained. Concerning the mechanism, although CS_2_ insertion by thiolate intermediates **T** to yield lithium trithiocarbonates, **TTC** was thermodynamically more favourable than S_8_ insertion to form polysulfide intermediates **PS**, propagation from the latter showed lower energy barriers in its reaction with PO (Figure 11b^2^).

Thereafter, a substantial improvement in scope and material properties was achieved through the terpolymerisation of PTA with S_8_ and a wide range of epoxides, again enabled by O/S exchange (Figure 11c).^[^ ^60^ ^]^ Poly(ester‐*alt*‐S_x_) with up to quantitative polymer and sequence selectivity (*M*_n,max_. = 33 kg mol^−1^, *Đ* = 1.7) could be prepared. This process tolerated a wide range of epoxides, including challenging monomers such as industrially relevant epichlorohydrin, geminally and vicinally disubstituted epoxides, and natural product‐derived vanillin glycidyl ether.

Capitalising on the large monomer scope the glass transition temperatures of the resulting polymers could be tuned from ca. −40 °C up to 130 °C. Furthermore, the good thermal stability of the polymers, enabled the thermal activation of the S_x_ links to insert additional S_8_ (Figure 10d). Even lipoic acid carrying carboxylic acid functionalities which would be intrinsically incompatible with the ROTERP methodology could be introduced via thermal insertion post‐polymerisation. Crosslinking the pendant aldehyde groups in the vanillin‐derived terpolymers with diamines produced mechanically robust and thermally processable covalent adaptable networks. These networks could be applied as adhesives for stainless steel and wood, whereas addition of lipoic acid into the network via sulphur–sulphur bond metathesis substantially improves adhesive performances on aluminum.

## 6 Sugar Derived Oxetanes

**Figure 12:**
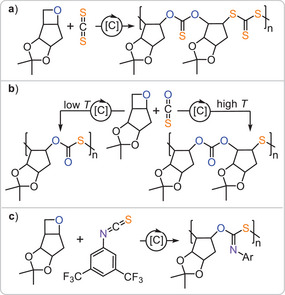
ROCOP of bicyclic Xylose derived oxetane with a) CS_2_, b) COS with and c) 3,5‐(CF_3_)_2_PhNCS.

Clearly, sulphur‐containing ROCOP and ROTERP are establishing themselves as useful methodologies. However, the monomers outlined above, including elemental sulphur itself, are petrochemically derived. Given the projected necessity of transitioning to bio‐based monomer feedstocks, achieving copolymerisation with such monomers is an intermediate requirement. In this context, bicyclic oxetanes can be obtained from acetal‐protected xylose in two synthetic steps. Turning these into sulphur‐containing copolymers (Figure 12), *Buchard* reported the ROCOP with CS₂, yielding a polymer with an poly(OSO‐*alt*‐SSS) microstructure in 95% sequence selectivity and 90% polymer selectivity (*M*_n,max_ = 14 kg mol^−1^, *Đ* = 1.8) using a SalcyCrCl/PPNCl bicomponent catalyst.^[^ ^61^ ^]^ Both temperature and catalyst choice drastically affected the selectivity, with OSS and thioether linkages also being observed. These polymers were UV‐degradable, and the degradation process was accelerated by the addition of silanes. *Wooley* and *Darensbourg* found that temperature‐controlled O/S scrambling could be achieved in SalcyCrCl/PPNCl‐catalysed COS copolymerisations.^[^ ^62^ ^]^ At 40 °C alternating poly(monothiocarbonates) (*M*_n_ = 3 kg mol^−1^, *Đ* = 1.2) featuring OSO links were produced. Increasing the reaction temperature to 120 °C yielded poly(carbonate‐*alt*‐thioethers) (*M*_n,max_ = 28 kg mol^−1^, *Đ* = 1.2). Compared to their oxygen‐only polycarbonate counterparts, the polymer produced by complete O/S scrambling substantially increased thermal stability achieving a *T*_d,onset_ of around 310 °C. This enhanced stability may result from the suppression of depolymerisation pathways to cyclic six‐membered heterocarbonates, with thioether groups intercepting the carbonate moieties, underscoring the potential for controlled scrambling to even enhance material properties. Relatedly, *Zhang* thereafter reported PPNCl/BEt_3_ bicomponent catalysis to facilitate alternating COS ROCOP without O/S scrambling at temperatures below 60 °C (*M*_n,max_ = 17 kg mol^−1^, *Đ* = 1.8).^[^ ^63^ ^]^ *Buchard* reported the ROCOP with 3,5‐bis(trifluoromethyl)‐substituted PhNCS under bicomponent aluminium trisphenolate/PPNCl catalysis, selectively yielding poly(monothioimidocarbonates) (*M*_n,max_ *_._* = 35 kg mol^−1^, *Đ* = 1.7). This polymerisation approach also allowed the ROCOP with aliphatic RNCS, terpolymerisations with bifunctional RNCS to produce crosslinked materials, and block polymers via either a macroinitiator or a stepwise monomer addition strategy.^[^ ^64^ ^]^

## 7 Conclusion and Outlook

In summary, sulphur‐containing ROCOP and ROTERP enables the efficient and selective formation of entirely new and complex polymer microstructures from relatively simple monomers through controlled O/S scrambling reactions. Key future objectives must prioritise achieving greater control over molar mass and chain‐ends, as none of the polymerisations presented above, despite being anionic, are strictly living. Broadened polydispersities, multimodal distributions, and significant deviations from theoretical molecular weights are common. Additionally, methodologies granting control over tacticity require further attention.^[^ ^65^, ^66^, ^67^, ^68^, ^69^, ^70^ ^]^ Achieving such control would not only maximise thermal and mechanical properties but also enable precise access to diverse polymer architectures for applications in, e.g., self‐assembly, elastomers, compatibilisers and more.^[^ ^71^, ^72^, ^73^, ^74^, ^75^ ^]^ At a more fundamental level, this requires a deeper understanding of how catalysis is affected by the transition from traditional oxygen‐based to sulphur‐containing monomers, as the discovery of sequence‐selective methodologies currently relies more on serendipity than rational development. In this regard many performant catalytic strategies for sulphur‐free polymerisations remain to be explored and understood in sulphur‐containing ROCOP and ROTERP.^[^ ^76^, ^77^, ^78^, ^79^, ^80^, ^81^ ^]^ Furthermore, insights on sequence selectivity generated in this filed could be leveraged to enable new polymerisations via cationic, radical or condensation methodologies.^[^ ^82^, ^83^, ^84^, ^85^, ^86^, ^87^ ^]^ Ultimately, this will allow for precise tailoring of polymer microstructures to meet the specific properties required for desired applications, as well as the combination of sulphur with all‐oxygen‐based polymers. As these materials are introduced to the market, it is important to recognise that directly competing with commodity plastics derived from petrochemical sources is likely to be difficult—if not unrealistic—at the outset. Therefore, to maximise the chances of commercial success, efforts should not be focused on replacing materials such as polyethylene for applications like plastic bags. Instead, the unique properties of sulphur should be leveraged in applications that cannot be easily achieved—or only with significant difficulty—without it. One promising example is the development of high‐refractive‐index polymers for use in optical lenses. Sulphur‐rich polymers can readily achieve refractive indices above 1.7, significantly exceeding the market standard, especially for materials that are compatible with injection molding. This enables the production of thinner lenses and provides greater design flexibility for optical components. In addition, the high affinity of sulphur for transition metals should be exploited in applications beyond the scavenging of contaminants for purification. For instance, this property could be used to facilitate the controlled formation of polymer–metal composites, enhancing thermal and electrical conductivity while preventing phase separation through favorable interactions between the polymer matrix and the metal particles. Other potential uses include the immobilization of metal catalysts or, in a more academic context, promoting polymer self‐assembly in solution via metal–ligand coordination. This could lead to the formation of catalytically active environments reminiscent of enzyme active sites. In commercial settings, sulphur's affinity for metals is also relevant for the development of coatings and sealants for metal components, offering added functionality and durability. Of course, the degradable nature of sulphur‐containing polymers can help alleviate some pollution concerns, particularly with regard to the accumulation of microplastics. Finally, it should be noted that not every application may require full sulphur incorporation in every repeat unit; in some cases, random or block copolymers may be more suitable for optimising performance, shelf‐life and processability.


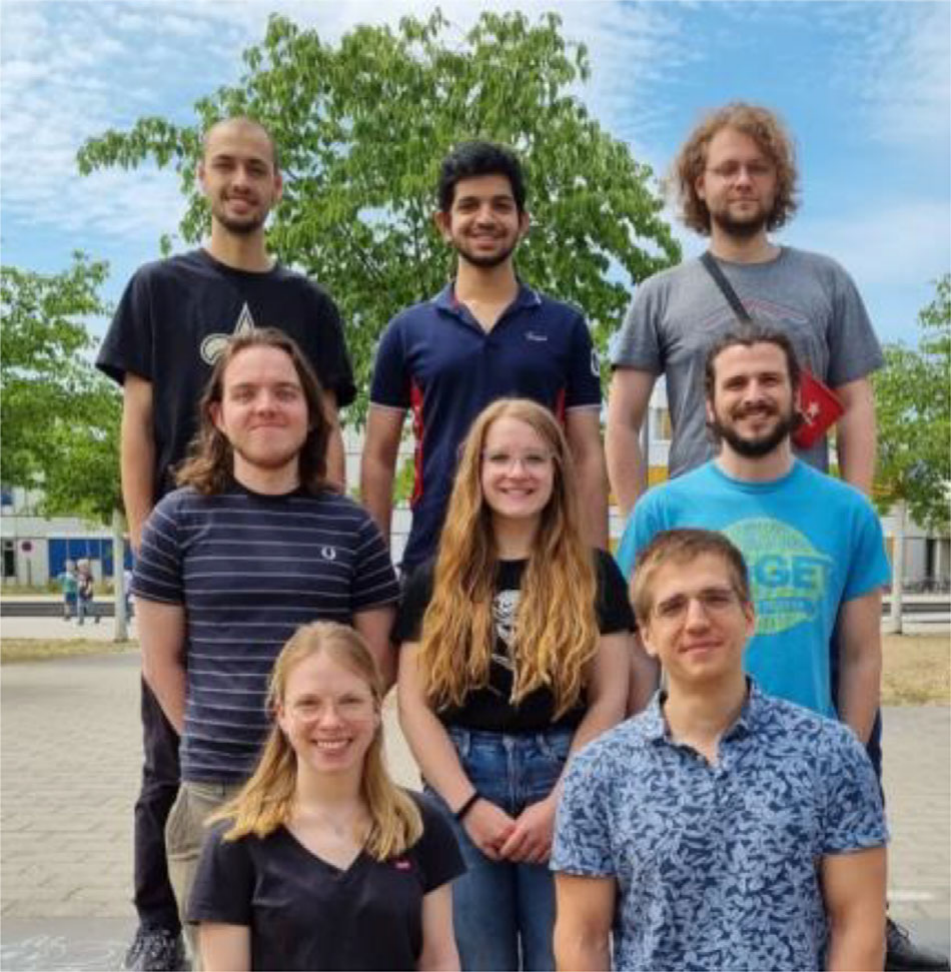


From top left to bottom right: M. R. Stühler (B.Sc. + M.Sc., FU Berlin; current joint Ph.D. with A. J. P.), B. R. Manjunatha (B.S./M.S., IISER Thiruvananthapuram; current Ph.D. with A. J. P.), D. Battke, C. Fornacon‐Wood (B.Sc., Sheffield; M.Sc., FU Berlin; current Ph.D. with A. J. P.), J. Stephan (B.Sc. + M.Sc., FU Berlin; current Ph.D. with A. J. P.), C. Gallizioli (B.Sc. + M.Sc., Pavia; current Ph.D. with A. J. P.), Marie Kreische, A. J. Plajer.

Alex J. Plajer is a Junior Professor (W1 tenure‐track to W3) at the University of Bayreuth. Born in Mannheim, Germany, he studied chemistry at Heidelberg University. He then completed an M.Phil. and Ph.D. degrees under the supervision of Dominic S. Wright at the University of Cambridge, supported by a Vice‐Chancellor's Scholarship. Following his doctorate, he was awarded a Royal Commission for the Exhibition of 1851 Fellowship at the University of Oxford. He subsequently secured a Liebig Fellowship at Freie Universität Berlin, where he established his independent research. In 2024, he joined Bayreuth as a tenure‐track Junior Professor, continuing his work on sustainable polymer materials, focusing on how sulphur (and other even more inorganic elements) shape catalysis, self‐assembly and material properties.

## Conflict of Interests

The authors declare no conflict of interest.

## Data Availability

Data sharing is not applicable to this article as no new data were created or analysed in this study.

## References

[1] H. Mutlu , E. B. Ceper , X. Li , J. Yang , W. Dong , M. M. Ozmen , P. Theato , Macromol. Rapid Commun. 2019, 40, 1800650. 10.1002/marc.201800650 30468540

[2] L. A. Limjuco , H. T. Fissaha , H. Kim , G. M. Nisola , W.‐J. Chung , ACS Appl. Polym. Mater. 2020, 2, 4677–4689.

[3] M. P. Crockett , A. M. Evans , M. J. H. Worthington , I. S. Albuquerque , A. D. Slattery , C. T. Gibson , J. A. Campbell , D. A. Lewis , G. J. L. Bernardes , J. M. Chalker , Angew. Chem. Int. Ed. 2016, 55, 1714–1718. 10.1002/anie.201508708 PMC4755153 26481099

[4] W. J. Chung , J. J. Griebel , E. T. Kim , H. Yoon , A. G. Simmonds , H. J. Ji , P. T. Dirlam , R. S. Glass , J. J. Wie , N. A. Nguyen , B. W. Guralnick , J. Park , Á. Somogyi , P. Theato , M. E. Mackay , Y.‐E. Sung , K. Char , J. Pyun , Nat. Chem. 2013, 5, 518–524. 23695634 10.1038/nchem.1624

[5] A. Napoli , M. Valentini , N. Tirelli , M. Müller , J. A. Hubbell , Nat. Mater. 2004, 3, 183–189. 14991021 10.1038/nmat1081

[6] J. J. Griebel , S. Namnabat , E. T. Kim , R. Himmelhuber , D. H. Moronta , W. J. Chung , A. G. Simmonds , K.‐J. Kim , J. van der Laan , N. A. Nguyen , E. L. Dereniak , M. E. Mackay , K. Char , R. S. Glass , R. A. Norwood , J. Pyun , Adv. Mater. 2014, 26, 3014–3018. 24659231 10.1002/adma.201305607

[7] D. H. Kim , W. Jang , K. Choi , J. S. Choi , J. Pyun , J. Lim , K. Char , S. G. Im , Sci. Adv. 2020, 6, eabb5320. 32923596 10.1126/sciadv.abb5320 PMC7455493

[8] X. Cao , H. Wang , J. Yang , R. Wang , X. Hong , X. Zhang , J. Xu , H. Wang , Chin. Chem. Let. 2022, 33, 1021–1024.

[9] G. W. Coates , Y. D. Y. L. Getzler , Nat. Rev. Mater. 2020, 5, 501–516.

[10] Y. Wang , M. Li , J. Chen , Y. Tao , X. Wang , Angew. Chem. Int. Ed. 2021, 60, 22547–22553. 10.1002/anie.202109767 34424604

[11] Y. Wang , Y. Zhu , W. Lv , X. Wang , Y. Tao , J. Am. Chem. Soc. 2023, 145, 1877–1885. 36594572 10.1021/jacs.2c11502

[12] K. A. Stellmach , M. K. Paul , M. Xu , Y.‐L. Su , L. Fu , A. R. Toland , H. Tran , L. Chen , R. Ramprasad , W. R. Gutekunst , ACS Macro Lett. 2022, 11, 895–901. 35786872 10.1021/acsmacrolett.2c00319

[13] S.‐Q. Wang , L.‐H. Liu , K. Li , W. Xiong , H.‐Z. Fan , Q. Cao , Z. Cai , J.‐B. Zhu , Polym. Chem. 2025, 16, 987–993.

[14] Y.‐L. Su , L. Yue , H. Tran , M. Xu , A. Engler , R. Ramprasad , H. J. Qi , W. R. Gutekunst , J. Am. Chem. Soc. 2023, 145, 13950–13956. 37307298 10.1021/jacs.3c03455 PMC10311534

[15] J. Yuan , W. Xiong , X. Zhou , Y. Zhang , D. Shi , Z. Li , H. Lu , J. Am. Chem. Soc. 2019, 141, 4928–4935. 30892027 10.1021/jacs.9b00031

[16] S. Mavila , B. T. Worrell , H. R. Culver , T. M. Goldman , C. Wang , C.‐H. Lim , D. W. Domaille , S. Pattanayak , M. K. McBride , C. B. Musgrave , C. N. Bowman , J. Am. Chem. Soc. 2018, 140, 13594–13598. 30351134 10.1021/jacs.8b09105

[17] S. Mavila , H. R. Culver , A. J. Anderson , T. R. Prieto , C. N. Bowman , Angew. Chem. Int. Ed. 2022, 134, e202110741. 10.1002/anie.202110741 34697873

[18] C. Shi , M. L. McGraw , Z.‐C. Li , L. Cavallo , L. Falivene , E. Y.‐X. Chen , Sci. Adv. 2020, 6, eabc0495. 32875116 10.1126/sciadv.abc0495 PMC7438104

[19] J. M. Scheiger , M. Hoffmann , P. Falkenstein , Z. Wang , M. Rutschmann , V. W. Scheiger , A. Grimm , K. Urbschat , T. Sengpiel , J. Matysik , M. Wilhelm , P. A. Levkin , P. Theato , Angew. Chem. Int. Ed. 2022, 61, e202114896. 10.1002/anie.202114896 PMC9302686 35068039

[20] J. Jia , J. Liu , Z.‐Q. Wang , T. Liu , P. Yan , X.‐Q. Gong , C. Zhao , L. Chen , C. Miao , W. Zhao , S. Cai , X.‐C. Wang , A. I. Cooper , X. Wu , T. Hasell , Z.‐J. Quan , Nat. Chem. 2022, 14, 1249–1257. 36302872 10.1038/s41557-022-01049-1

[21] S. J. Tonkin , C. T. Gibson , J. A. Campbell , D. A. Lewis , A. Karton , T. Hasell , J. M. Chalker , Chem. Sci. 2020, 11, 5537–5546. 32874497 10.1039/d0sc00855a PMC7441575

[22] J. Herzberger , K. Niederer , H. Pohlit , J. Seiwert , M. Worm , F. R. Wurm , H. Frey , Chem. Rev. 2016, 116, 2170–2243. 26713458 10.1021/acs.chemrev.5b00441

[23] A. J. Plajer , C. K. Williams , Angew. Chem. Int. Ed. 2022, 61, e202104495. 10.1002/anie.202104495 PMC9298364 34015162

[24] N. Adachi , Y. Kida , K. Shikata , J. Polym. Sci.: Polym. Chem. 1977, 15, 937–944.

[25] Y. Sun , C. Zhang , X. Zhang , Chem. ‐ Eur. J. 2024, 30, e202401684. 38802324 10.1002/chem.202401684

[26] D. J. Darensbourg , S. J. Wilson , A. D. Yeung , Macromolecules 2013, 46, 8102–8110.

[27] M. Luo , X.‐H. Zhang , D. J. Darensbourg , Macromolecules 2015, 48, 5526–5532.

[28] J. Diebler , H. Komber , L. Häußler , A. Lederer , T. Werner , Macromolecules 2016, 49, 4723–4731.

[29] J. Stephan , M. R. Stühler , S. M. Rupf , S. Neale , A. J. Plajer , Cell Rep. Phys. Sci. 2023, 4, 101510.

[30] G.‐W. Yang , R. Xie , Y.‐Y. Zhang , C.‐K. Xu , G.‐P. Wu , Chem. Rev. 2024, 124, 12305–12380. 39454031 10.1021/acs.chemrev.4c00517

[31] M. Luo , X.‐H. Zhang , B.‐Y. Du , Q. Wang , Z.‐Q. Fan , Macromolecules 2013, 46, 5899–5904.

[32] M. R. Stühler , M. Kreische , C. Fornacon‐Wood , S. M. Rupf , R. Langer , A. J. Plajer , Chem. Sci. 2024, 15, 19029–19036. 39479163 10.1039/d4sc05858e PMC11515943

[33] L.‐Y. Wang , G.‐G. Gu , T.‐J. Yue , W.‐M. Ren , X.‐B. Lu , Macromolecules 2019, 52, 2439–2445.

[34] L.‐Y. Wang , G.‐G. Gu , B.‐H. Ren , T.‐J. Yue , X.‐B. Lu , W.‐M. Ren , ACS Catal. 2020, 10, 6635–6644.

[35] X.‐L. Chen , B. Wang , D.‐P. Song , L. Pan , Y.‐S. Li , Macromolecules 2022, 55, 1153–1164.

[36] K. Han , M. Wang , Z. Ding , Z. Zhou , B. Wang , Y. Li , Polym. Chem. 2024, 15, 2502–2512.

[37] M. R. Stühler , C. Gallizioli , S. M. Rupf , A. J. Plajer , Polym. Chem. 2023, 14, 4848–4855.

[38] B. R. Manjunatha , M. R. Stühler , L. Quick , A. J. Plajer , Chem. Commun. 2024, 60, 4541–4544. 10.1039/d4cc00811a 38497828

[39] C. Fornacon‐Wood , B. R. Manjunatha , M. R. Stühler , C. Gallizioli , C. Müller , P. Pröhm , A. J. Plajer , Nat. Commun. 2023, 14, 4525. 37500621 10.1038/s41467-023-39951-y PMC10374558

[40] D. J. Darensbourg , A. I. Moncada , W. Choi , J. H. Reibenspies , J. Am. Chem. Soc. 2008, 130, 6523–6533. 18444619 10.1021/ja800302c

[41] D. J. Darensbourg , P. Ganguly , W. Choi , Inorg. Chem. 2006, 45, 3831–3833. 16676934 10.1021/ic052109j

[42] G. Feng , X. Feng , X. Liu , W. Guo , C. Zhang , X. Zhang , Macromolecules 2023, 56, 6798–6805.

[43] C. Fornacon‐Wood , M. R. Stühler , C. Gallizioli , B. R. Manjunatha , V. Wachtendorf , B. Schartel , A. J. Plajer , Chem. Commun. 2023, 59, 11353–11356. 10.1039/d3cc03315e 37655470

[44] B. R. Manjunatha , K. S. Marcus , R. M. Gomila , A. Frontera , A. J. Plajer , Green Chem. 2025, 27, 3494–3502.

[45] M. Luo , X.‐H. Zhang , D. J. Darensbourg , Acc. Chem. Res. 2016, 49, 2209–2219. 27676451 10.1021/acs.accounts.6b00345

[46] X.‐F. Zhu , G.‐W. Yang , R. Xie , G.‐P. Wu , Angew. Chem. Int. Ed. 2022, 61, e202115189. 10.1002/anie.202115189 34866295

[47] X.‐F. Zhu , R. Xie , G.‐W. Yang , X.‐Y. Lu , G.‐P. Wu , ACS Macro Lett. 2021, 10, 135–140. 35548986 10.1021/acsmacrolett.0c00831

[48] L. Song , M. Liu , D. You , W. Wei , H. Xiong , Macromolecules 2021, 54, 10529–10536.

[49] X.‐F. Zhu , X.‐Y. Lu , C.‐K. Xu , Y.‐B. Fang , G.‐W. Yang , W. Li , J. Wang , G.‐P. Wu , Chin. J. Chem. 2023, 41, 3311–3318.

[50] J. Stephan , J. L. Olmedo‐Martínez , C. Fornacon‐Wood , M. R. Stühler , M. Dimde , D. Braatz , R. Langer , A. J. Müller , H. Schmalz , A. J. Plajer , Angew. Chem. Int. Ed. 2024, 63, e202405047. 10.1002/anie.202405047 38520388

[51] J. Stephan , M. R. Stühler , C. Fornacon‐Wood , M. Dimde , K. Ludwig , H. Sturm , J. L. Olmedo‐Martínez , A. J. Müller , A. J. Plajer , Polym. Chem. 2025, 16, 1003–1009.

[52] C. A. L. Lidston , B. A. Abel , G. W. Coates , J. Am. Chem. Soc. 2020, 142, 20161–20169. 33176426 10.1021/jacs.0c10014

[53] S. Rupf , P. Pröhm , A. J. Plajer , Chem. Sci. 2022, 13, 6355–6365. 35733883 10.1039/d2sc01776h PMC9159086

[54] P. Deglmann , S. Machleit , C. Gallizioli , S. M. Rupf , A. J. Plajer , Cat. Sci.Tech. 2023, 13, 2937–2945.

[55] D. Silbernagl , H. Sturm , A. J. Plajer , Polym. Chem. 2022, 13, 3981–3985.

[56] A. J. Plajer , ChemCatChem 2022, 14, e202200867.

[57] S. Penczek , R. Ślazak , A. Duda , Nature 1978, 273, 738–739.

[58] J.‐Y. Chao , T.‐J. Yue , B.‐H. Ren , G.‐G. Gu , X.‐B. Lu , W.‐M. Ren , Angew. Chem. Int. Ed. 2022, 61, e202115950. 10.1002/anie.202115950 35129257

[59] C. Gallizioli , D. Battke , H. Schlaad , P. Deglmann , A. J. Plajer , Angew. Chem. Int. Ed. 2024, 63, e202319810. 10.1002/anie.202319810 38421100

[60] C. Gallizioli , P. Deglmann , A. J. Plajer , Angew. Chem. Int. Ed. 2025, e202501337. 10.1002/anie.202501337 PMC12281093 40208780

[61] T. M. McGuire , A. Buchard , Polym. Chem. 2021, 12, 4253–4261.

[62] D. K. Tran , A. N. Braaksma , A. M. Andras , S. K. Boopathi , D. J. Darensbourg , K. L. Wooley , J. Am. Chem. Soc. 2023, 145, 18560–18567. 37578470 10.1021/jacs.3c05529 PMC10863053

[63] G. Feng , X. Feng , X. Liu , C. Zhang , X. Zhang , Macromolecules 2024, 57, 3757–3764.

[64] E. F. Clark , G. Kociok‐Köhn , M. G. Davidson , A. Buchard , Polym. Chem. 2023, 14, 2838–2847.

[65] T.‐J. Yue , Y. Xiao , B.‐H. Ren , X.‐B. Lu , W.‐M. Ren , J. Am. Chem. Soc. 2025, 147, 3607–3614. 39825845 10.1021/jacs.4c15343

[66] T.‐J. Yue , W.‐M. Ren , L. Chen , G.‐G. Gu , Y. Liu , X.‐B. Lu , Angew. Chem. Int. Ed. 2018, 130, 12852–12856. 10.1002/anie.201805200 30088310

[67] Y. Zhu , M. Li , Y. Wang , X. Wang , Y. Tao , Angew. Chem. Int. Ed. 2023, 62, e202302898. 10.1002/anie.202302898 37058315

[68] K. Li , J.‐L. Cheng , M.‐Y. Wang , W. Xiong , H.‐Y. Huang , L.‐W. Feng , Z. Cai , J.‐B. Zhu , Angew. Chem. Int. Ed. 2024, 63, e202405382. 10.1002/anie.202405382 38682252

[69] X.‐Y. Fu , T.‐J. Yue , X.‐H. Guo , X.‐B. Lu , W.‐M. Ren , Nat. Commun. 2025, 16, 2154. 40038273 10.1038/s41467-025-57449-7 PMC11880438

[70] C. E. Brubaker , D. Velluto , D. Demurtas , E. A. Phelps , J. A. Hubbell , ACS Nano 2015, 9, 6872–6881. 26125494 10.1021/acsnano.5b02937

[71] K.‐S. Kang , A. Phan , C. Olikagu , T. Lee , D. A. Loy , M. Kwon , H. Paik , S. J. Hong , J. Bang , W. O. Parker, Jr. , M. Sciarra , A. R. de Angelis , J. Pyun , Angew. Chem. Int. Ed. 2021, 60, 22900–22907. 10.1002/anie.202109115 34402154

[72] J.‐Z. Zhao , T.‐J. Yue , B.‐H. Ren , X.‐B. Lu , W.‐M. Ren , Nat. Commun. 2024, 15, 3002. 38589410 10.1038/s41467-024-47382-6 PMC11001992

[73] L. Zhou , L. T. Reilly , C. Shi , E. C. Quinn , E. Y.‐X. Chen , Nat. Chem. 2024, 16, 1357–1365. 38649467 10.1038/s41557-024-01511-2

[74] G.‐W. Yang , Y.‐Y. Zhang , G.‐P. Wu , Acc. Chem. Res. 2021, 54, 4434–4448. 34806374 10.1021/acs.accounts.1c00620

[75] X. Geng , X. Liu , Q. Yu , C. Zhang , X. Zhang , J. Am. Chem. Soc. 2024, 146, 25852–25859. 39226029 10.1021/jacs.4c09394

[76] A. Hermann , S. Hill , A. Metz , J. Heck , A. Hoffmann , L. Hartmann , S. Herres‐Pawlis , Angew. Chem. Int. Ed. 2020, 59, 21778–21784. 10.1002/anie.202008473 PMC7814670 32954634

[77] H. Li , J. Ollivier , S. M. Guillaume , J.‐F. Carpentier , Angew. Chem. Int. Ed. 2022, 61, e202202386. 10.1002/anie.202202386 35286752

[78] W. T. Diment , W. Lindeboom , F. Fiorentini , A. C. Deacy , C. K. Williams , Acc. Chem. Res. 2022, 55, 1997–2010. 35863044 10.1021/acs.accounts.2c00197 PMC9350912

[79] W. Gruszka , J. A. Garden , Nat. Commun. 2021, 12, 3252. 34059676 10.1038/s41467-021-23192-y PMC8167082

[80] R. A. Smith , G. Fu , O. McAteer , M. Xu , W. R. Gutekunst , J. Am. Chem. Soc. 2019, 141, 1446–1451. 30636410 10.1021/jacs.8b12154

[81] P. Yuan , Y. Sun , X. Xu , Y. Luo , M. Hong , Nat. Chem. 2022, 14, 294–303. 34824460 10.1038/s41557-021-00817-9

[82] L. Chen , R. Hu , B. Z. Tang , J. Am. Chem. Soc. 2025, 147, 1134–1146. 39707976 10.1021/jacs.4c14708

[83] Y. Xia , T. Shao , Y. Sun , J. Wang , C. Gu , C. Zhang , X. Zhang , Nat. Commun. 2025, 16, 1974. 40000662 10.1038/s41467-025-57208-8 PMC11862183

[84] H. Huang , S. Zheng , J. Luo , L. Gao , Y. Fang , Z. Zhang , J. Dong , N. Hadjichristidis , Angew. Chem. Int. Ed. 2024, 63, e202318919. 10.1002/anie.202318919 38169090

[85] S. Wang , Z.‐Y. Tian , H. Lu , Angew. Chem. Int. Ed. 2024, 63, e202411630. 10.1002/anie.202411630 39073287

[86] N. M. Bingham , Z. Abousalman‐Rezvani , K. Collins , P. J. Roth , Polym. Chem. 2022, 13, 2880–2901.

[87] A. W. Woodhouse , A. Kocaarslan , J. A. Garden , H. Mutlu , Macromol. Rapid Commun. 2024, 45, 2400260. 10.1002/marc.202400260 38824417

